# From Fetal Growth Restriction to Adolescent Cardiometabolic Risk: The Impact of Catch-Up Growth and Adiposity

**DOI:** 10.3390/nu18050843

**Published:** 2026-03-05

**Authors:** Anca Adam-Raileanu, Alin Horatiu Nedelcu, Mitica Ciorpac, Carmen Rodica Anton, Ancuta Lupu, Laura Bozomitu, Lorenza Forna, Sorana Caterina Anton, Costica Mitrofan, Ionela Daniela Morariu, Emil Anton, Dragos Munteanu, Elena Cristina Mitrofan, Vasile Valeriu Lupu

**Affiliations:** 1 Grigore T. Popa University of Medicine and Pharmacy, 700115 Iasi, Romania; anca.adam-raileanu@umfiasi.ro (A.A.-R.); alin.nedelcu@umfiasi.ro (A.H.N.); mitica.ciorpac@umfiasi.ro (M.C.); carmen.anton@umfiasi.ro (C.R.A.); lorenza.forna@umfiasi.ro (L.F.); sorana.anton@umfiasi.ro (S.C.A.); costel_mitrofan@yahoo.com (C.M.); ionela.morariu@umfiasi.ro (I.D.M.); emil.anton@umfiasi.ro (E.A.); dragos.munteanu@umfiasi.ro (D.M.); vasile.lupu@umfiasi.ro (V.V.L.); 2 CF Clinical Hospital, 700506 Iasi, Romania; criselend@yahoo.com

**Keywords:** fetal growth restriction, catch-up growth, cardiometabolic risk, adolescent

## Abstract

**Background/Objectives:** Fetal growth restriction (FGR) represents a model of adverse intrauterine programming associated with an increased risk of cardiometabolic disorders later in life. We examined the relationships between birth weight, catch-up growth, adipokine signaling, and early cardiometabolic risk in adolescents. **Methods**: This cross-sectional study included 80 term-born adolescents (40 FGR, 40 controls) matched for age and sex. Anthropometry, blood pressure, lipid profile, fasting glucose, adipokines (leptin, adiponectin), and ghrelin levels were assessed. Associations between birth weight, growth rate, adipokines, and cardiometabolic outcomes were analyzed. **Results**: Birth weight was not associated with adiposity, lipid profile, blood pressure, or glycemic status (*p* > 0.05). In contrast, catch-up growth in the FGR group was correlated with increased BMI (ρ = 0.680, *p* < 0.001), central adiposity (ρ = 0.714, *p* < 0.001), systolic blood pressure (ρ = 0.448, *p* = 0.0037) and diastolic blood pressure (ρ = 0.325, *p* = 0.0409). Mediation analyses showed that the current BMI largely explains the associations between catch-up growth and cardiometabolic risk, systolic blood pressure, and waist circumference (β = 2.832 kg/m^2^ per 1-unit increase in ΔZ; *p* < 0.001). The hypertensive effect of catch-up growth was amplified in overweight/obese adolescents (β = 8.13 mmHg; *p* = 0.006). Catch-up growth was independently associated with higher leptin (β = 220 ng/L; *p* = 0.022) and a higher leptin/ghrelin ratio (β = 2.330; *p* = 0.034). **Conclusions:** Postnatal growth acceleration, rather than fetal size alone, drives early cardiometabolic susceptibility following FGR through adiposity-mediated and endocrine pathways.

## 1. Introduction

Fetal growth restriction (FGR), defined as the failure of a fetus to achieve its genetically determined growth potential, affects a substantial proportion of pregnancies worldwide and remains a major public health concern [1]. Beyond its well-established association with increased perinatal morbidity and mortality, accumulating evidence suggests that FGR confers long-term vulnerability to non-communicable medical conditions, particularly cardiometabolic illnesses, later in life [2,3]. This concept is based on Barker’s observations linking low birth weight with higher rates of coronary heart disease, type 2 diabetes, and hypertension in adulthood, forming the basis for the hypothesis of developmental origins of health and disease (DOHaD) [4,5,6].

According to the DOHaD framework, adverse conditions during critical periods of fetal development induce permanent structural, functional, and metabolic adaptations that enhance short-term survival, but may predispose individuals to morbid clinical states when postnatal environmental settings differ from those anticipated in utero [7,8]. In the context of FGR, reduced nutrient and oxygen supply leads to adaptive fetal responses aimed at preserving vital organ function, including altered glucose–insulin metabolism, redistribution of blood flow, and changes in endocrine signaling [9,10,11]. Although these adaptations are advantageous for fetal survival, they can result in long-lasting metabolic programming that increases susceptibility to cardiovascular and metabolic disease [12,13].

### 1.1. Fetal Metabolic Programming and Endocrine Adaptations

FGR is characterized by a complex interplay of placental insufficiency, altered nutrient availability, and hormonal dysregulation [14,15]. Experimental and clinical studies have demonstrated that growth-restricted fetuses exhibit reduced insulin sensitivity, impaired pancreatic β-cell development, altered hypothalamic–pituitary–adrenal (HPA) axis activity, and changes in adipose tissue development [16,17,18,19]. These endocrine adaptations are believed to “program” metabolic pathways toward energy conservation, favoring fat accumulation and insulin resistance in later life [20,21].

In addition to classical endocrine axes, increasing attention has been directed toward the brain–gut–hormone axis as a potential mediator linking fetal growth disturbances to long-term metabolic impairments [22,23]. Hormones such as leptin, ghrelin, and adiponectin play key roles in appetite regulation, energy homeostasis, and insulin sensitivity [24]. Disruption of these endocrine signaling pathways during critical developmental windows may permanently alter central and peripheral mechanisms controlling food intake, adiposity, and metabolic regulation [25,26].

### 1.2. Catch-Up Growth: From Physiological Adaptation to Pathological Risk

Postnatal growth trajectories critically modulate the long-term consequences of FGR [27,28]. Catch-up growth, defined as accelerated postnatal weight gain following intrauterine growth restriction, is often considered a desirable and physiological process that allows children to approach their genetically determined growth potential [29]. However, growing evidence suggests that rapid or excessive postnatal recovery growth may shift from a compensatory mechanism to a pathological process [30].

Accelerated catch-up growth has been associated with increased adiposity, central fat accumulation, insulin resistance, and elevated blood pressure, especially when occurring in nutritionally abundant postnatal environments [31,32,33,34]. This phenomenon has been described as a “mismatch” between the nutrient-scarce intrauterine environment and the nutrient-rich postnatal milieu, amplifying the adverse metabolic consequences of fetal programming [3]. Importantly, not all individuals born with FGR experience adverse outcomes, highlighting the pivotal role of postnatal growth patterns and postnatal nutrition in shaping the risk of metabolic syndrome and cardiovascular disease [35].

### 1.3. Metabolic Syndrome in Adolescence: An Early Window of Risk

Cardiometabolic risk is defined as the presence and clustering of metabolic abnormalities—including central adiposity, dyslipidemia, elevated blood pressure, and impaired glucose metabolism—that increase the likelihood of cardiovascular disease and type 2 diabetes [36]. These elements reflect shared pathophysiological mechanisms, including insulin resistance, chronic low-grade inflammation, endothelial dysfunction, and ectopic fat accumulation. Metabolic syndrome is not synonymous, but a clinical expression of cardiometabolic risk, being increasingly recognized in pediatric populations [37]. Although traditionally viewed as an adult disorder, evidence indicates that the pathophysiological processes underlying MetS often originate in childhood and adolescence [38]. Early manifestations of cardiometabolic dysfunction during adolescence are particularly concerning, as they track strongly into adulthood and are associated with premature cardiovascular disease [39,40].

Adolescence represents a critical developmental window marked by profound hormonal, metabolic, and body composition changes. Puberty-related insulin resistance, changes in fat distribution, and rising blood pressure may unmask latent vulnerabilities established during fetal life [41,42]. Evaluating the probability of developing cardiometabolic disease during adolescence therefore provides a unique opportunity to identify early deviations from healthy metabolic trajectories, particularly in vulnerable populations such as individuals born with FGR.

### 1.4. Adipokines as Emerging Biomarkers of Cardiometabolic Risk

Adipose tissue is now recognized as an active endocrine organ that secretes a wide range of bioactive molecules, collectively termed adipokines. Two adipokines, leptin and adiponectin, and ghrelin—a circulating peptide hormone synthesized predominantly by gastric cells—play central roles in regulating energy balance, appetite, insulin sensitivity, and inflammation [43]. Alterations in adipokine and gut-derived hormone secretion and signaling have been implicated in the development of obesity, insulin resistance, hypertension, and dyslipidemia [44].

In individuals with a history of FGR, dysregulated endocrine regulators of energy balance such as leptin, ghrelin and adiponectin profiles may reflect early disturbances in adipose tissue function and serve as sensitive biomarkers of cardiometabolic risk before the onset of overt illness [23]. Ratios such as leptin/adiponectin or leptin/ghrelin have been proposed as integrative markers capturing the balance between pro- and anti-metabolic signaling pathways [43,45]. However, data on adipokine profiles in adolescents born with FGR, particularly in relation to catch-up growth and current nutritional status, remain limited and inconsistent [46].

### 1.5. Novelty and Significance of the Study

While the association between low birth weight and adult cardiometabolic pathology is well established, critical gaps remain regarding when metabolic and cardiovascular potential for adverse outcomes first becomes detectable and which postnatal factors drive risk expression in individuals with a history of FGR. Most existing studies, both on human and animal models, focus on adult outcomes, rely on birth weight alone as a proxy for fetal adversity, or fail to disentangle the effects of fetal growth from postnatal growth trajectories [47,48,49,50]. Moreover, data integrating catch-up growth, adiposity distribution, blood pressure regulation, and adipokine signaling during adolescence are scarce.

The present study addresses these gaps by providing a comprehensive assessment of cardiometabolic risk in early adolescence, a developmental window in which fetal programming effects may begin to emerge but before overt disease becomes established. By jointly examining birth weight, longitudinal catch-up growth, current nutritional status, and adipokine profiles, this work aims to move beyond a static view of fetal size, highlighting the dynamic interplay between early growth patterns and contemporary metabolic risk.

## 2. Materials and Methods

### 2.1. Study Design

This was an observational, cross-sectional study with prospective recruitment. Participants were evaluated at a single time point. Our study’s main objective was to assess the relative contributions of birth weight, postnatal catch-up growth and current adiposity to early cardiometabolic risk in adolescents antenatally affected by FGR, with a specific focus on adipokine signaling and its role in mediating associations between growth trajectories and cardiometabolic outcomes.

### 2.2. Participants

The study included children recruited from Saint Mary Children Hospital in Iași, Romania, from 1 January 2024 to 30 June 2025. Participants were divided into two groups: 40 children with a documented history of FGR and 40 healthy children, with similar age, sex and BMI distribution. All children were aged between 10 and 15 years old and were born at term.

The FGR children group was defined by children with a documented medical history of FGR, without associated neonatal impairments, congenital malformations or history of twin pregnancy, chronic use of hypoglycemic, lipid-modifying, or antihypertensive medications, or strict dietary restrictions that may interfere with metabolic markers. Formal informed consent provided by a legal guardian was mandatory for inclusion in the study.

The Control group was defined by children born with appropriate-for-gestational-age (AGA) birth weight, with no evidence of neonatal pathologies, a history of twin pregnancy, congenital malformations, chronic medical entities or circumstances known to affect growth or nutritional status. Written informed consent from a legal guardian was required for participants to be enrolled in the study.

Following enrollment in the study, each patient provided a detailed medical history and underwent a comprehensive clinical examination conducted by a physician. The following variables were recorded: demographic data (age, sex, and living environment—urban or rural); perinatal characteristics (birth weight, gestational age, and mode of delivery); feeding pattern during the first six months of life (exclusive breastfeeding, formula feeding, or mixed feeding); associated comorbidities; current anthropometric parameters (height, weight, body mass index, waist circumference, and waist-to-height ratio); blood pressure values; fasting blood glucose concentration; and serum lipid profile, including triglycerides, total cholesterol, and cholesterol fractions (LDL, HDL, and VLDL cholesterol).

Morning blood samples (7:00–9:00 a.m.) were obtained from each participant for the measurement of serum total ghrelin, leptin, and adiponectin using commercially available enzyme-linked immunosorbent assay (ELISA) kits (R&D Systems Inc., Minneapolis, MN, USA).

### 2.3. Anthropometric and Clinical Variables and Term Definitions

Standardized measurements were used to determine the current nutritional status and clinical variables:

- **Weight and Height**: Used to calculate BMI (weight (kg) divided by height squared (m^2^)) and corresponding *Z*-scores based on WHO reference curves, adjusted for age and sex [51]. According to the WHO interpretation of BMI for age (5 to 19 years) chart, a normal weight was defined as a BMI value between −2 and +2 SD, overweight was defined as a BMI value between +1 SD and ≤+2 SD, and obesity was defined as a BMI value of >+2 SD. Catch-up growth was defined as a rise greater than 0.67 in weight- or BMI-for-age *z*-scores relative to birth or current measurements. This 0.67 cutoff was used because it reflects the distance between successive percentile lines on commonly used pediatric growth charts, thus representing a clinically meaningful shift across percentile ranges [52].
- **Abdominal Circumference (WC):** Used to identify central obesity, defined as WC > 90 percentile, adjusted for age and sex [53].
- **Waist-to-Height Ratio (WHtR**): Calculated to assess abdominal adiposity, with a threshold of WHtR ≥ 0.5 → abdominal obesity [54].
- **Blood Pressure:** Systolic and diastolic values will be categorized by age and sex percentiles according to the National High Blood Pressure Education Program guidelines [55,56].

Term definitions:

- **Metabolic syndrome (MetS):** According to the International Diabetes Federation (IDF) pediatric criteria, metabolic syndrome in children and adolescents aged 10 to <16 years is defined by the presence of central obesity, assessed by waist circumference ≥ the 90th percentile, in addition to at least two of the following criteria: triglyceride levels ≥ 1.7 mmol/L (≥1500 mg/L), HDL cholesterol < 1.03 mmol/L (<400 mg/L), blood pressure ≥130/85 mmHg or ≥the 90th percentile for age, sex, and height, and fasting plasma glucose ≥ 5.6 mmol/L (≥1000 mg/L) or previously diagnosed type 2 diabetes [54].
- **Dyslipidemia:** According to the Expert Panel on Integrated Guidelines for Cardiovascular Health [57], for children aged 10–19 years, borderline high values for total cholesterol are considered to be between 1700 and 1990 mg/L, and high values are defined as ≥2000 mg/L; LDL cholesterol values between 1100 and 1290 mg/L are considered borderline high, while a value ≥ 1300 mg/L is considered high; HDL values < 400 mg/L are considered low; triglyceride values between 900 and 1290 mg/L are considered borderline high, while a value ≥ 1300 mg/L represents a high value [57].
- **Term birth** is defined as delivery occurring between 37 + 0 and 41 + 6 completed weeks of gestation [58].
- **Appropriate for gestational age (AGA)** refers to neonates whose birth weight lies between the 10th and 90th percentiles for gestational age and sex, based on standardized population reference charts [59,60,61].

### 2.4. Ethical Considerations

Ethical approval for the conduct of this study was granted by the Ethics Committee of the Grigore T. Popa University of Medicine and Pharmacy, Iași, Romania (No. 383/27 January 2024), as well as by the Ethics Committee of Saint Mary Children’s Hospital, Iași (No. 31602/26 October 2023).

### 2.5. Statistical Analysis

Statistical analyses were performed using R software (version 3.4; R Foundation for Statistical Computing, Vienna, Austria). Continuous variables were assessed for normality using visual inspection of histograms and the Shapiro–Wilk test. As most continuous variables showed non-normal distributions, results are presented as median and interquartile range (IQR) or mean ± standard deviation, as appropriate. Categorical variables are reported as counts and percentages.

Comparisons between the Control and FGR groups were conducted using the Wilcoxon rank-sum test for continuous variables and the Chi-square test or Fisher’s exact test for categorical variables, depending on expected cell counts. Associations between continuous variables were evaluated using Spearman’s rank correlation coefficient (ρ).

Multivariable linear regression models were used to examine independent associations between birth weight parameters, catch-up growth (ΔZ), and continuous cardiometabolic endpoints. All regression models were adjusted for age and sex, and additional interaction terms were included where appropriate to assess effect modification by current weight status (normal weight vs. overweight/obesity).

For binary cardiometabolic outcomes with sufficient event counts, multivariable logistic regression models were constructed. Predictor selection was guided by LASSO regularization to reduce multicollinearity and overfitting, followed by parsimonious adjusted models. Due to the low prevalence of hypertension, Firth penalized logistic regression was used as a sensitivity analysis to obtain bias-reduced estimates.

To investigate potential mechanistic pathways, causal mediation analyses were performed in the FGR group using current BMI as a mediator in the relationship between catch-up growth and selected cardiometabolic outcomes. Mediation models were adjusted for age and sex, and indirect (ACME), direct (ADE), and total effects and the proportion mediated were estimated using non-parametric bootstrap resampling with 2000 simulations. All statistical tests were two-sided, and a *p*-value < 0.05 was considered statistically significant.

## 3. Results

### 3.1. Baseline Characteristics and Current Nutritional Status

The baseline demographic, perinatal, early-life, and current nutritional characteristics of the study population are summarized in Table 1. The Control and FGR groups were comparable with respect to sex distribution (*p* = 0.805), age (mean 13.5 ± 1.5 years; median 14 years; *p* > 0.05), environmental conditions (urban vs. rural; *p* = 0.644), mode of delivery (natural birth vs. cesarean section; *p* = 0.251), and type of alimentation during the first six months of life (breastfed, formula-fed, mixed alimentation; *p* = 1.000).

In line with the study design, anthropometric measures did not differ between groups. Body mass index (BMI), BMI *z*-score, waist circumference, and waist-to-height ratio were similar in the Control and FGR groups (*p* = 0.733, *p* = 0.593, *p* = 0.847, and *p* = 0.203, respectively), as was the distribution of weight status according to World Health Organization criteria, including the prevalence of overweight and obesity (*p* = 0.605). Collectively, these findings support good baseline comparability between the Control and FGR groups, limiting the potential influence of selection bias on subsequent analyses.

Birth weight was not associated with current BMI or BMI *z*-score in the overall sample (BMI: ρ = −0.01, *p* = 0.910; BMI *z*-score: ρ = 0.01, *p* = 0.896). Stratified analyses yielded consistent results, with no significant correlations observed in either the Control group (BMI: ρ = −0.27, *p* = 0.090; BMI *z*-score: ρ = −0.21, *p* = 0.196) or the FGR group (BMI: ρ = 0.04, *p* = 0.788; BMI *z*-score: ρ = 0.03, *p* = 0.875).

### 3.2. Association Between Birth Weight and Metabolic Syndrome Elements

Birth weight showed no significant association with most components of metabolic syndrome in the study population (Table 2). Specifically, no statistically significant correlations were observed between birth weight (absolute value or *z*-score) and markers of abdominal obesity (waist circumference, waist-to-height ratio) or dyslipidemia, including serum triglycerides, total cholesterol, HDL cholesterol, and LDL cholesterol, either in the overall cohort or when analyses were stratified by study group. These findings were further confirmed by multivariable linear regression analyses adjusted for age, sex, and study group, which demonstrated no independent associations between birth weight and measures of abdominal adiposity or lipid profile. Similarly, no associations were identified between birth weight and blood pressure, including systolic and diastolic values or percentile-based classifications.

Regarding glycemic status, birth weight was not significantly correlated with fasting glucose levels in univariate analyses. Multivariable linear regression analyses adjusted for age, sex, and study group showed a borderline association between birth weight and glucose levels (β = 232 mg/L per kg, *p* = 0.057), with comparable results when birth weight was expressed as a *z*-score (β = 116 mg/L per *z*-score unit, *p* = 0.057). Elevated glycemia (high glucose) was rare in the study population (2/80, 2.5%) and both cases were found in our Control group. However, elevated glycemia prevalence did not differ significantly between the Control and FGR groups (*p* = 0.494).

### 3.3. Association Between Birth Weight and Adipokine Profile

In the overall study population, birth weight showed a borderline inverse correlation with leptin levels (ρ = −0.19, *p* = 0.092). Similarly, borderline inverse correlations were observed between birth weight and ghrelin levels (ρ = −0.20, *p* = 0.071) and between birth weight and the leptin/adiponectin ratio (ρ = −0.20, *p* = 0.071). No significant correlation was identified between birth weight and adiponectin levels (ρ = 0.07, *p* = 0.526). Stratified analyses within the Control and FGR groups showed consistent directions for leptin, without reaching statistical significance (Table 3A).

In multivariable linear regression analyses adjusted for age, sex, and study group, birth weight was not independently associated with adiponectin, ghrelin, or adipokine ratios. A borderline inverse association between birth weight and leptin persisted after adjustment (β = −888 ng/L per kg, *p* = 0.074; equivalent results for birth weight *z*-score: β = −0.444, *p* = 0.074). Interaction analyses did not indicate effect modification by study group for the association between birth weight and leptin (birth weight × group interaction, *p* = 0.887). In the same model, age was positively associated with leptin levels (*p* = 0.0048), while sex showed a borderline association (*p* = 0.062)—Table 3B.

### 3.4. Association Between Catch-Up Growth and Cardiometabolic Risk in the FGR Group

Catch-up growth was defined as a change in weight-for-age *Z*-score between birth and the study visit of more than 0.67 SD (ΔZ = Z.aW − Z.bW). In the FGR group (*n* = 40), the median ΔZ was 1.38 (IQR 1.78) and the mean ΔZ was 1.50 ± 1.38. Using ΔZ > 0.67 SD as the operational definition, 34/40 children (85.0%) exhibited catch-up growth, whereas 6/40 (15.0%) showed no catch-up.

In categorical comparisons (Table 4A), children with catch-up growth had a higher prevalence of overweight/obesity than those without catch-up (41.2% vs. 0%), although this difference did not reach statistical significance (Fisher’s exact *p* = 0.074). No significant associations were identified between catch-up status and abdominal obesity (*p* = 1.000), WC ≥ P90 (*p* = 0.567), WHtR ≥ 0.5 (*p* = 1.000), high glucose (*p* = 1.000), elevated systolic BP ≥ P90 (*p* = 1.000), or elevated diastolic BP ≥ P90 (*p* = 0.493).

In continuous comparisons (Table 4B), children with catch-up growth had significantly higher current BMI (median 21.570 vs. 16.815 kg/m^2^; *p* = 0.020) and BMI *Z*-score (median 0.530 vs. −1.015; *p* = 0.005). Waist circumference and WHtR did not differ significantly by catch-up status (WC: *p* = 0.116; WHtR: *p* = 0.459), and no significant differences were observed for blood pressure, lipid profile, fasting glucose, or adipokine ratios in these unadjusted group comparisons (all *p* > 0.05).

**Figure 1:**
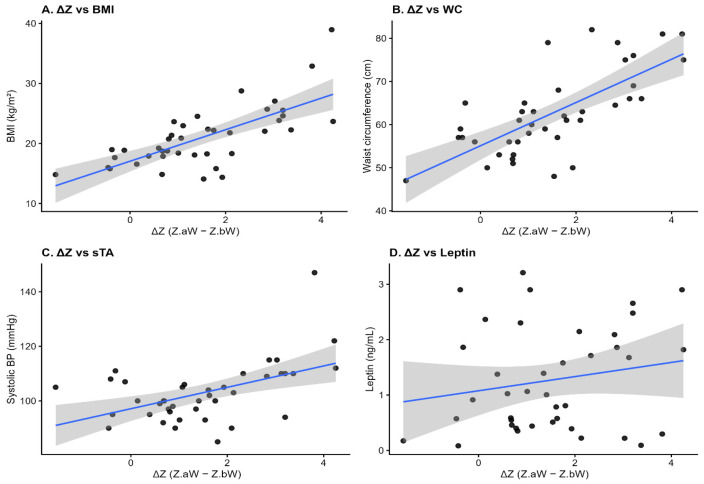
Catch-up growth (ΔZ) and selected cardiometabolic outcomes in the FGR group. Scatterplots display individual values with fitted linear regression lines and 95% confidence intervals (*n* = 40).

When ΔZ was treated as a continuous exposure, Spearman analyses demonstrated strong positive correlations with BMI (ρ = 0.680, *p* < 0.001), BMI *Z*-score (ρ = 0.740, *p* < 0.001), waist circumference (ρ = 0.714, *p* < 0.001), and WHtR (ρ = 0.504, *p* < 0.001). ΔZ also correlated positively with systolic BP (ρ = 0.448, *p* = 0.0037), diastolic BP (ρ = 0.325, *p* = 0.0409), and systolic BP percentile (ρ = 0.344, *p* = 0.0297), whereas no significant correlations were found with lipid parameters, fasting glucose, or adiponectin (all *p* > 0.05). Associations between ΔZ and leptin or the leptin/ghrelin ratio were not significant in correlation analyses (*p* = 0.427 and *p* = 0.217, respectively). These relationships are illustrated in Figure 1.

In multivariable linear regression adjusted for age and sex (Table 4C), ΔZ remained independently associated with higher BMI (β = 2.832 kg/m^2^ per 1-unit increase in ΔZ; *p* < 0.001), BMI *Z*-score (β = 0.713; *p* < 0.001), waist circumference (β = 5.334 cm; *p* < 0.001), and WHtR (β = 0.022; *p* = 0.003). ΔZ was also independently associated with higher systolic BP (β = 4.113 mmHg; *p* < 0.001), diastolic BP (β = 2.836 mmHg; *p* = 0.021), and systolic BP percentile (β = 6.997; *p* = 0.024), while the association with diastolic BP percentile did not reach statistical significance (*p* = 0.066). Among adipokine measures, ΔZ was independently associated with higher leptin (β = 220 ng/L; *p* = 0.022) and a higher leptin/ghrelin ratio (β = 2.330; *p* = 0.034).

Finally, in adjusted logistic regression, ΔZ was associated with increased odds of WC ≥ P90 (OR = 4.198 per 1-unit increase in ΔZ; 95% CI 1.400–12.587; *p* = 0.010), whereas other binary outcomes could not be reliably modeled due to insufficient event counts—Table 4D.

### 3.5. Interaction Between Catch-Up Growth and Current Weight Status in the FGR Group

To further investigate whether the cardiometabolic impact of catch-up growth differed according to current weight status, interaction analyses between catch-up growth (ΔZ) and overweight/obesity (OWOB) were performed in the FGR group (*n* = 40). At the time of evaluation, 26 children (65%) had normal weight, while 14 (35%) were classified as overweight or obese.

In multivariable linear regression models adjusted for age and sex, significant main effects of ΔZ were observed among children with normal weight. In this subgroup, ΔZ was positively associated with BMI *Z*-score (β = 0.43 per 1-unit ΔZ; *p* = 0.005) and waist circumference (β = 2.16 cm; *p* = 0.034, Table 5), indicating that greater catch-up growth was associated with increased overall and central adiposity even in the absence of overt overweight.

No statistically significant ΔZ × OWOB interactions were observed for BMI, BMI *Z*-score, waist circumference, WHtR, lipid parameters, glucose, or adipokines, although point estimates for anthropometric outcomes tended to be larger among overweight/obese children. However, a statistically significant interaction between ΔZ and OWOB was identified for systolic blood pressure. The interaction term (ΔZ × OWOB) was positively associated with systolic blood pressure (β = 8.13 mmHg; *p* = 0.006), indicating that the effect of catch-up growth on systolic blood pressure was substantially stronger in children with overweight or obesity compared with those of normal weight. Stratified analyses supported this finding, showing no significant association between ΔZ and systolic blood pressure in normal-weight children (β = −0.47 mmHg; *p* = 0.72), whereas a strong positive association was observed in overweight/obese children (β = 7.17 mmHg; *p* = 0.044).

**Figure 2:**
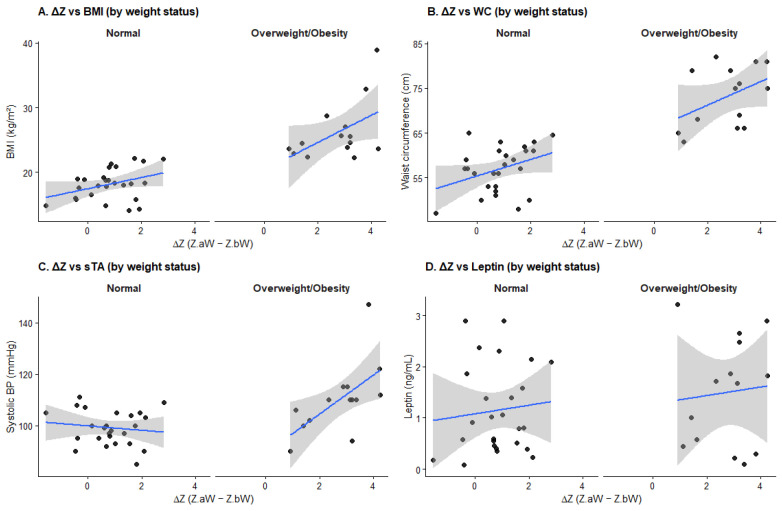
Interaction between catch-up growth and current weight status in the FGR group. Scatterplots depict the associations between catch-up growth (ΔZ > 0.67SD; ΔZ = Z.aW − Z.bW) and (**A**) BMI, (**B**) waist circumference, (**C**) systolic blood pressure, and (**D**) leptin concentration, stratified by current weight status (normal weight vs. overweight/obesity). Solid lines represent fitted linear regression lines with 95% confidence intervals. Models are adjusted for age and sex (*n* = 40).

As illustrated in Figure 2**,** the analysis reveals that the intensity of catch-up growth has a differentiated impact on cardiometabolic outcomes based on current weight status. While normoponderal children maintain stable physiological markers, those in the overweight/obese (OWOB) category display divergent, steeper slopes for BMI, waist circumference, and systolic blood pressure (Figure 2A–C).

Furthermore, Figure 2D demonstrates that higher weight acceleration is associated with elevated leptin levels primarily in the OWOB group, suggesting that rapid postnatal recovery, when coupled with current excess weight, significantly drives adiposity-related hormonal dysfunction and early cardiovascular risk.

### 3.6. Independent Predictors of Cardiometabolic Risk

In the final multivariable model for dyslipidemia (35 events among 80 children), neither adipokine ratios nor age emerged as independent predictors. Specifically, the leptin/ghrelin ratio (OR = 0.80; 95% CI 0.36–1.02; *p* = 0.383), leptin/adiponectin ratio (OR = 0.10; 95% CI 0.00–1.18; *p* = 0.303), and age (OR = 0.86; 95% CI 0.62–1.18; *p* = 0.348) were not significantly associated with the presence of dyslipidemia—Table 6.

**Figure 3:**
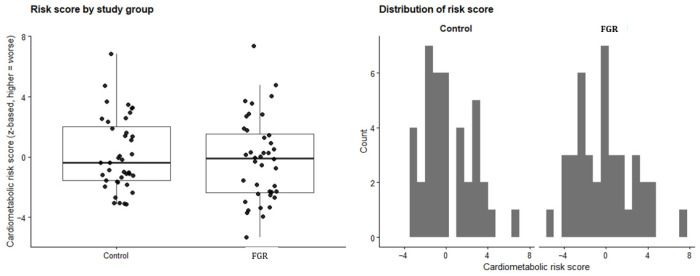
Distribution of the cardiometabolic risk score according to study group.

For elevated blood pressure (seven events), current BMI was identified as the only independent predictor. Each 1 kg/m^2^ increase in BMI was associated with 28% higher odds of elevated blood pressure (OR = 1.28; 95% CI 1.09–1.60; *p* = 0.007), independent of age and sex. Age was not significantly associated with elevated blood pressure in the adjusted model (OR = 0.72; 95% CI 0.40–1.29; *p* = 0.253)—Figure 3. No other cardiometabolic outcomes (abdominal obesity, obesity status, or hyperglycemia) could be reliably modeled due to a low number of events and were therefore excluded from multivariable analysis.

### 3.7. Mediation Analysis of the Relationship Between Catch-Up Growth and Cardiometabolic Risk

**Figure 4:**
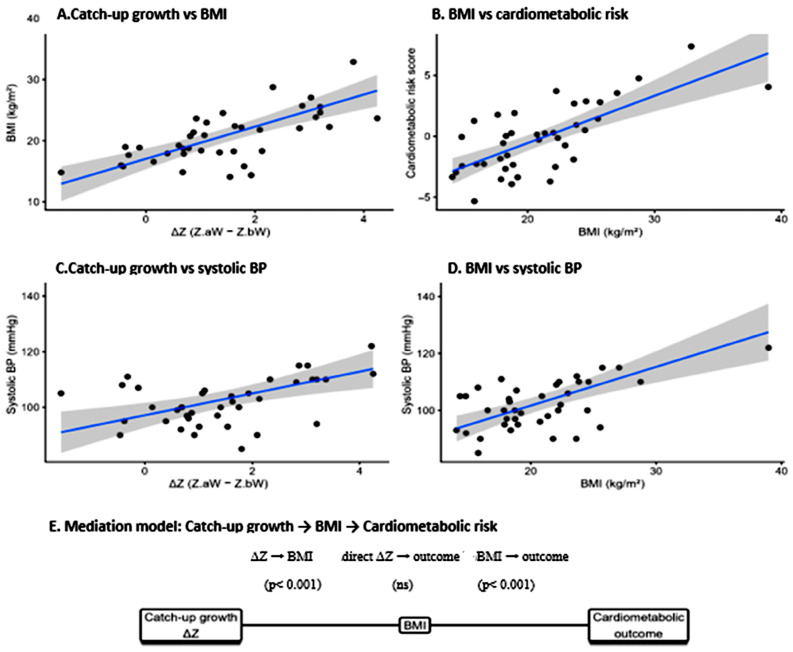
Mediation of the association between catch-up growth and cardiometabolic outcomes by current BMI in the FGR group. (**A**–**D**) Scatterplots showing the associations between catch-up growth (ΔZ), BMI, cardiometabolic risk score, and systolic blood pressure. Lines represent fitted linear regression models with 95% confidence intervals. (**E**) Conceptual path diagram illustrating the mediation model.

Mediation analyses were conducted in the FGR group (*n* = 40) to explore whether current BMI may account for the association between catch-up growth (ΔZ) and cardiometabolic outcomes, adjusting for age and sex. ΔZ was associated with the composite cardiometabolic risk score (total effect = 1.08, *p* = 0.004). The indirect pathway through BMI was significant (ACME = 1.15, *p* < 0.001), whereas the direct effect of ΔZ was not significant after accounting for BMI (ADE = −0.07, *p* = 0.802)—Figure 4.

For systolic blood pressure, ΔZ showed a significant total effect (total effect = 4.11 mmHg, *p* = 0.003), with evidence of mediation through BMI (ACME = 3.55 mmHg, *p* = 0.053) and no significant direct effect (ADE = 0.57 mmHg, *p* = 0.704). For waist circumference, ΔZ was strongly associated with central adiposity (total effect = 5.33 cm, *p* < 0.001). This association was largely explained by BMI (ACME = 4.02 cm, *p* < 0.001), while a borderline direct effect persisted (ADE = 1.32 cm, *p* = 0.065). Collectively, these findings support BMI as a key intermediate factor linking catch-up growth to adiposity-related cardiometabolic risk in adolescents with a history of FGR.

### 3.8. Sensitivity Analysis—Firth Logistic Regression

**Figure 5:**
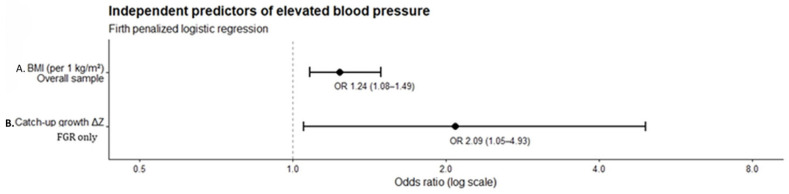
Forest plot of predictors of elevated blood pressure using Firth penalized logistic regression. The plot shows odds ratios (OR) and 95% confidence intervals for (**A**) current BMI predicting elevated blood pressure in the overall cohort (*n* = 80) and (**B**) catch-up growth (ΔZ) predicting elevated blood pressure in the FGR subgroup (*n* = 40). Models are adjusted for age and sex.

Sensitivity analyses using Firth penalized logistic regression were performed to account for the low number of hypertension events (Figure 5). In the overall study population (*n* = 80), current BMI remained the only independent predictor of elevated blood pressure (OR = 1.24 per 1 kg/m^2^ increase; 95% CI 1.08–1.49; *p* = 0.003), while study group (FGR vs. Control), age, and sex were not significantly associated with hypertension. In analyses restricted to the FGR group (*n* = 40; 5 events), catch-up growth quantified as ΔZ was independently associated with elevated blood pressure (OR = 2.09 per 1-unit increase in ΔZ; 95% CI 1.05–4.93; *p* = 0.036), whereas age and sex were not significant predictors. These findings support the robustness of the main results and indicate that the cardiometabolic impact of catch-up growth on blood pressure is specific to children with a history of intrauterine growth restriction.

## 4. Discussion

### 4.1. Absence of Global Cardiometabolic Differences Between FGR and Control Adolescents

One of the main findings of the present study is the absence of significant differences between adolescents with a documented history of FGR and their AGA peers with respect to current nutritional status, lipid profile, fasting glycemia, blood pressure, and the composite cardiovascular and metabolic risk. Despite the well-established association between impaired fetal growth and cardiometabolic disease in adulthood, our report indicates that FGR *per se* does not inevitably translate into an adverse cardiometabolic phenotype during early adolescence, particularly in the absence of excess adiposity or other postnatal metabolic stressors.

These findings are consistent with the DOHaD concept, which emphasizes that FGR confers increased susceptibility to later cardiovascular pathology and metabolic disorders rather than early clinical expression of underlying predisposing factors [24]. Longitudinal cohort studies further suggest that cardiometabolic sequelae of low birth weight often remain subclinical during childhood and adolescence and tend to emerge in adulthood, particularly in the presence of accelerated postnatal growth or obesity [13].

Several pediatric studies support the absence of overt metabolic and cardiovascular disturbances in children and adolescents born small for gestational age (SGA) or with FGR. Nordman et al. reported no independent association between birth size and lipid profile, blood pressure, or insulin resistance during childhood after adjustment for current body size [39], while Chakraborty et al. found no increase in obesity or metabolic abnormalities at 9 years of age among children with FGR who experienced appropriate postnatal growth [62]. These results align with our observation of comparable anthropometric and metabolic characteristics between FGR and control adolescents. This is further supported by the low and similar prevalence of dyslipidemia, metabolic syndrome, obesity, abdominal obesity, and overweight in both groups.

Consistent with this interpretation, birth weight was not associated with current anthropometric parameters in our cohort. Neither BMI nor BMI Z-score correlated with birth weight in the overall sample or within study groups. Similar observations have been reported by Baran et al. [63], who showed that birth weight and length were weak predictors of overweight and obesity in children aged 4–15 years, and by Bettiol et al. [64], who demonstrated that FGR increased adult BMI risk primarily in individuals who developed overweight during childhood [63,64]. These data underscore the dominant role of postnatal growth trajectories over fetal size alone.

Regarding individual cardiometabolic components, no significant associations were observed between birth weight and markers of abdominal adiposity, lipid profile, or blood pressure, consistent with large pediatric cohort studies reporting weak or absent correlations once current body size is considered [65,66]. Although modest blood pressure elevations have been described in some populations born with FGR [65,67], these effects are generally small and more evident later in life or under circumstances of rapid postnatal weight gain.

Fasting glucose levels were also largely comparable between groups, with only a borderline association between birth weight and glycemia in multivariable models. This finding aligns with studies suggesting that subtle impairments in glucose metabolism may precede overt dysglycemia in individuals born SGA. Jornayvaz et al., for example, reported reduced whole-body glucose oxidation in such children despite normal fasting glucose levels [68]. The low prevalence of impaired fasting glucose in our cohort likely limited the detection of these early metabolic changes.

Overall, these findings support the notion that the risk of cardiometabolic disorders associated with FGR is not uniformly expressed during childhood or adolescence but is strongly modulated by postnatal factors, particularly excess weight gain and adiposity. Adult reports demonstrate that low birth weight is associated with increased visceral adiposity and insulin resistance primarily in individuals who become overweight later in life [47,49], and that the combination of low birth weight and childhood obesity markedly amplifies cardiometabolic disease potential [42].

### 4.2. Catch-Up Growth as a Key Determinant of Cardiometabolic Risk Within the FGR Group

In contrast to birth weight, the magnitude of catch-up growth—defined as a change in weight-for-age *Z*-score (ΔZ) greater than 0.67 SD—emerged as a strong determinant of cardiometabolic risk within the FGR group. ΔZ showed robust positive connections with BMI, BMI *Z*-score, waist circumference, waist-to-height ratio, systolic and diastolic blood pressure, and systolic blood pressure percentile, independent of age and sex. These findings indicate that accelerated postnatal growth, rather than intrauterine growth restriction itself, is the primary driver of adverse cardiometabolic profiles in adolescents affected by FGR.

This pattern is consistent with human and longitudinal studies demonstrating preferential fat mass accretion and persistent increases in adiposity following early catch-up growth after intrauterine growth impairments [32,35,69]. Beltrand et al. showed that infants with FGR undergoing catch-up growth rapidly restored fat mass, even in the absence of early metabolic abnormalities [35]. In our cohort, the lack of association between ΔZ and dyslipidemia or fasting glucose suggests that alterations in adiposity and blood pressure may precede the development of overt metabolic pathological conditions.

Mechanistic plausibility is provided by the “thrifty catch-up fat” hypothesis, which proposes that suppressed thermogenesis during postnatal refeeding favors disproportionate fat storage and predisposes individuals to later obesity and insulin resistance [33,70]. Research models further support this notion, demonstrating persistent modifications in adipose tissue insulin signaling, adipogenesis, and energy metabolism following catch-up growth after FGR [71,72].

Beyond adiposity, the strong association between ΔZ and blood pressure highlights the relevance of catch-up growth for early cardiovascular programming. Epidemiological studies have shown increased cardiovascular mortality in adults born SGA who experienced rapid childhood growth [73], and pediatric cohorts similarly report higher blood pressure in children with accelerated postnatal growth following FGR [74,75]. Proposed mechanisms include reduced nephron endowment, increased arterial stiffness, and long-lasting changes in vascular structure and function induced by rapid growth after intrauterine undernutrition [29,76].

Notably, despite its strong associations with adiposity and blood pressure, catch-up growth was not associated with dyslipidemia or fasting glucose in our adolescent cohort. This pattern suggests a hierarchical progression of cardiometabolic risk, whereby early alterations in body composition and blood pressure precede overt disturbances in lipid and glucose metabolism. Human metabolic studies support this interpretation, showing that children born SGA may exhibit early insulin resistance or altered insulin secretion despite normal fasting glucose levels [77,78].

### 4.3. Role of Adiposity and Interaction with Current Weight Status

A major finding of the present study is that the cardiometabolic impact of catch-up growth is strongly modified by current weight status. Although ΔZ was associated with increased adiposity even among normal-weight adolescents, its effect on systolic blood pressure was markedly amplified in individuals with overweight or obesity. This interaction supports a “two-hit” model of cardiometabolic programming in which early growth acceleration induces biological susceptibility, while excess adiposity acts as a second hit that amplifies cardiovascular risk [79,80].

Stratified analyses confirmed that the association between ΔZ and systolic blood pressure was minimal in normal-weight adolescents but pronounced among those with overweight or obesity. Multivariable and sensitivity analyses consistently identified current BMI as the strongest independent predictor of elevated blood pressure. These findings are consistent with epidemiological studies showing that birth weight-related differences in blood pressure become clinically relevant primarily in the presence of increased weight gain [65,66,81,82].

With a synergistic effect of low birth weight and obesity on cardiometabolic risk, Stinson et al. demonstrated that adolescents exposed to both low birth weight and current obesity exhibited the highest risk profiles, whereas low birth weight in the absence of obesity conferred minimal vulnerability [42]. This interaction aligns with reports of U-shaped associations between birth weight and blood pressure, in which both low and high birth weight increase risk, particularly when accompanied by excess adiposity [83].

From a mechanistic perspective, excess adiposity may exacerbate blood pressure elevations through multiple pathways, including sympathetic activation, insulin resistance, low-grade inflammation, and adipokine imbalance. Obesity-related adjustments in leptin and adiponectin signaling have been implicated in pediatric hypertension, particularly in children with underlying developmental vulnerability such as FGR [84,85]. Available studies further suggest that FGR induces permanent alterations in renal, vascular, and neuroendocrine regulation that may remain clinically silent until challenged by increased adiposity [67,86,87].

### 4.4. Mechanistic Insights from Mediation Analysis and Clinical Implications

Causal mediation analyses demonstrated that the association between catch-up growth and the composite cardiometabolic risk was almost entirely explained by current BMI rather than birth size itself. A similar pattern was observed for systolic blood pressure, with near-complete mediation by BMI in both our report and other several studies [79,88,89]. These findings indicate that increased postnatal adiposity represents the principal mechanistic link between early growth acceleration and adolescent cardiovascular and metabolic disorders.

For waist circumference, BMI mediated approximately three-quarters of the total effect of ΔZ, while a borderline direct effect persisted, suggesting that additional mechanisms—such as preferential visceral fat accumulation or endocrine dysregulation—may contribute to central adiposity beyond overall body mass. Previous studies have reported disproportionate visceral fat deposition and altered adipose tissue function in individuals born growth-restricted who undergo catch-up growth, even after adjustment for BMI [22,75,90]. Experimental data further support long-lasting changes in adipose tissue distribution and adipokine signaling following FGR and rapid postnatal growth, changes which are not fully captured by BMI alone [71,74,91].

From a clinical perspective, these findings emphasize that while catch-up growth is often desirable in children born with FGR, its magnitude and quality warrant careful monitoring. Early nutritional and lifestyle interventions aimed at promoting healthy growth patterns and preventing excessive fat accumulation may be critical in mitigating long-term cardiometabolic disease risk in this vulnerable population [28,30,92].

### 4.5. Adipokine Profile and Early Metabolic Signaling

Although no statistically significant associations were observed between birth weight and adipokines after adjustment, borderline inverse relationships with leptin, ghrelin, and the leptin/adiponectin ratio suggest subtle variations in adipose tissue signaling. Similar modest associations between fetal growth parameters and circulating adipokines have been reported in pediatric and young adult cohorts [93,94].

Leptin remained weakly but consistently inversely associated with birth weight after adjustment, supporting the theory that fetal growth may influence long-term adipose tissue function and leptin regulation. Altered leptin signaling has been reported in children born SGA and in those undergoing catch-up growth. Correspondingly, preclinical evidence suggests that early growth restriction may predispose to leptin resistance, particularly when followed by rapid postnatal growth [95,96,97,98].

However, these associations were modest and did not differ by study group, indicating that adipokine dysregulations alone are unlikely to represent the primary pathway linking FGR to cardiometabolic vulnerability in adolescence. Rather, circulating leptin, ghrelin, and adiponectin levels appear to be strongly influenced by current nutritional status, pubertal stage, and adiposity, often outweighing the effects of birth characteristics [99,100,101]. Ghrelin and adiponectin play key roles in appetite regulation, adipocyte lipid accumulation, and insulin sensitivity, suggesting that early modifications in these pathways may reflect adaptive mechanisms that predispose to later metabolic vulnerability without immediate clinical expression [102,103,104]. Once again, longitudinal data indicate that such adaptations may become clinically relevant only when compounded by excess adiposity or adverse lifestyle factors later in life [46,97].

### 4.6. Limitations and Future Directions

Several limitations of the present study should be acknowledged. First, the relatively modest sample size, particularly within the FGR subgroup (*n* = 40), yielded low event rates for cardiometabolic outcomes—hyperglycemia (*n* = 0), abdominal obesity (*n* = 7, 17.5%), high SBP (*n* = 2, 5%), and high DBP (*n* = 4, 10%)—rendering these rare outcomes that limited statistical power for detecting associations. As a result, some associations, especially those involving binary endpoints, should be interpreted with caution despite the use of appropriate sensitivity analyses, including Firth penalized logistic regression. Second, the cross-sectional design precludes causal inference regarding the temporal relationships between catch-up growth, adiposity, and cardiometabolic outcomes. Although mediation analyses provide valuable mechanistic insight, they rely on assumptions that cannot be fully verified in observational data. Longitudinal follow-up from early childhood into adulthood would be necessary to confirm the observed pathways and to determine whether the cardiometabolic imbalances identified in adolescence persist or progress later in life.

Third, catch-up growth was quantified using changes in weight-for-age *Z*-scores, which does not allow differentiation between lean mass and fat mass accretion. Future studies incorporating detailed body composition assessment, such as dual-energy X-ray absorptiometry or bioimpedance analysis, would help clarify whether specific patterns of tissue gain are responsible for the observed cardiometabolic effects.

In addition, although several relevant confounders were accounted for, including age and sex, residual confounding related to pubertal stage, dietary intake, physical activity, socioeconomic status, or genetic susceptibility cannot be excluded. The absence of pubertal staging data is particularly relevant given the profound hormonal and metabolic changes occurring during adolescence.

Future research should therefore prioritize larger, longitudinal cohorts of individuals born with intrauterine growth restriction, with repeated assessments of growth trajectories, body composition, and cardiometabolic health across the life course. Such studies would allow evaluation of critical windows during which catch-up growth may be most harmful, as well as identification of modifiable factors that could mitigate long-term negative consequences. From a clinical perspective, intervention studies focusing on optimizing the quality—not merely the magnitude—of postnatal growth may provide important insights into preventive strategies for cardiometabolic disease in this vulnerable population.

## 5. Conclusions

FGR alone does not confer increased cardiometabolic risk in early adolescence. Instead, the magnitude of postnatal growth acceleration—particularly when accompanied by overweight or obesity—emerges as the principal determinant of adiposity and elevated blood pressure in this population. Current BMI acts as both a central mediator and an independent predictor linking early growth trajectories to adolescent cardiometabolic risk. Subtle shifts in adipokine signaling associated with postnatal growth acceleration suggest early endocrine adaptations that may contribute to later metabolic vulnerability. These findings support a conditional model of fetal programming, in which early growth restriction increases susceptibility that is unmasked by excessive postnatal weight gain. Clinically, they underscore the importance of monitoring not only growth recovery but also growth quality. Early nutritional and lifestyle interventions aimed at preventing excessive fat accumulation may reduce the probability of progression toward adult metabolic syndrome in individuals antenatally affected by growth restriction.

## Figures and Tables

**Table 1:** Baseline demographic, perinatal, nutritional, and metabolic characteristics of the study population according to study group.

Variable	Category	Control (N = 40) *n* (%)	FGR (N = 40) *n* (%)	*p*-Value	Test
**Sex**	Female	28 (70.0)	29 (72.5)	0.805	Chi-square
	Male	12 (30.0)	11 (27.5)		
**Environmental** **conditions**	Rural	24 (60.0)	26 (65.0)	0.644	Chi-square
	Urban	16 (40.0)	14 (35.0)		
**Type of birth**	Natural	27 (67.5)	22 (55.0)	0.251	Chi-square
	Cesarean	13 (32.5)	18 (45.0)		
**Alimentation in the first 6 months**	Breastfed	25 (62.5)	24 (60.0)	1.000	Fisher
	Formula-fed	12 (30.0)	12 (30.0)		
	Mixed	3 (7.5)	4 (10.0)		
**Weight status (WHO)**	Normal	28 (70.0)	26 (65.0)	0.605	Fisher
	Overweight	12 (30.0)	12 (30.0)		
	Obesity	0 (0.0)	2 (5.0)		
**Metabolic syndrome**	Yes	0 (0.0)	1 (2.5)	1.000	Fisher
	No	40 (100.0)	39 (97.5)		
**Abdominal obesity** **(WC > P90)**	Yes	6 (15)	7 (17.5)	1.000	Fisher
	No	34 (85)	33 (82.5)		
**Dyslipidemia**	Normal	22 (55.0)	23 (57.5)	0.822	Chi-square
	Hypertriglyceridemia and borderline high	18 (45.0)	17 (42.5)		
	Mixed dyslipidemia	0 (0.0)	0 (0.0)		
	Hypercholesterolemia and borderline high	11 (27.5)	16 (40)		

Data are presented as number (percentage). *p*-values were calculated using the Chi-square test or Fisher’s exact test, as appropriate. WHO, World Health Organization; FGR, intrauterine growth restriction.

**Table 2:** Association between birth weight parameters and metabolic syndrome elements.

Category	Outcome Parameter	Birth Weight Parameter	Group	*n*	Correlation (ρ)	*p*-Value
**Abdominal Obesity**	**Waist circumference (cm)**	Birth weight (kg)	Overall	80	−0.10	0.384
			Control	40	−0.24	0.130
			FGR	40	−0.08	0.639
	**WHtR**	Birth weight (kg)	Overall	80	−0.18	0.101
			Control	40	−0.14	0.373
			FGR	40	−0.10	0.550
**Glycemia**	**Glucose (mg/L)**	Birth weight (kg)	Overall	80	−0.00	0.970
			Control	40	0.16	0.319
			FGR	40	0.10	0.541
**Lipid Profile**	**Triglycerides (mg/L)**	Birth weight (kg)	Overall	80	0.03	0.821
			Control	40	0.21	0.204
			FGR	40	−0.02	0.905
	**Total cholesterol (mg/L)**	Birth weight (kg)	Overall	80	−0.12	0.298
			Control	40	−0.27	0.089
			FGR	40	0.16	0.327
	**HDL cholesterol (mg/L)**	Birth weight (kg)	Overall	80	0.00	0.973
	**LDL cholesterol (mg/L)**	Birth weight (kg)	Overall	80	−0.07	0.556
**Blood Pressure**	**sBP (mmHg)**	Birth weight (kg)	Overall	80	0.11	0.323
			Control	40	−0.00	0.999
			FGR	40	0.14	0.382
	**dBP (mmHg)**	Birth weight (kg)	Overall	80	0.13	0.257
	**P.sBP**	Birth weight (kg)	Overall	80	0.10	0.371
	**P.dBP**	Birth weight (kg)	Overall	80	0.09	0.433

Data are presented as Spearman’s correlation coefficients (ρ) or number, as appropriate. Linear regression models were adjusted for age, sex, and study group. *p*-values for categorical comparisons were calculated using Fisher’s exact test where appropriate.

**Table 3:** Association between birth weight parameters and adipokine profile. (**A**) Spearman correlations (overall and by study group); (**B**) multivariable linear regression (adjusted for age, sex, and study group).

(**A**)					
**Group**	**Birth Weight Parameter**	**Adipokine/Ratio**	***n***	**ρ**	***p*-Value**
**Overall**	Birth weight (kg)	Leptin (ng/L)	80	−0.19	0.092
**Control**	Birth weight (kg)	Leptin (ng/L)	40	−0.24	0.135
**FGR**	Birth weight (kg)	Leptin (ng/L)	40	−0.21	0.202
**Overall**	Birth weight (kg)	Adiponectin (µg/L)	80	0.07	0.526
**Overall**	Birth weight (kg)	Ghrelin (ng/L)	80	−0.20	0.071
**Overall**	Birth weight (kg)	Leptin/adiponectin ratio	80	−0.20	0.071
**Overall**	Birth weight z-score	Leptin (ng/L)	80	−0.19	0.092
**Overall**	Birth weight z-score	Adiponectin (µg/L)	80	0.07	0.526
**Overall**	Birth weight z-score	Ghrelin (ng/L)	80	−0.20	0.071
**Overall**	Birth weight z-score	Leptin/adiponectin ratio	80	−0.20	0.071
(**B**)					
**Outcome**	**Predictor**		**β**	**SE**	***p*-Value**
**Leptin (ng/L)**	Birth weight (kg)		−0.89	0.49	0.074
**Adiponectin (µg/L)**	Birth weight (kg)		0.12	5.69	0.984
**Ghrelin (ng/L)**	Birth weight (kg)		0.40	1.87	0.832
**Leptin/adiponectin ratio**	Birth weight (kg)		0.65	1.64	0.695
**Leptin/ghrelin ratio**	Birth weight (kg)		−1.20	3.74	0.750
**Leptin (ng/L)**	Birth weight z-score		−0.44	0.25	0.074
**Adiponectin (µg/L)**	Birth weight z-score		0.06	2.85	0.984
**Ghrelin (ng/L)**	Birth weight z-score		0.20	0.94	0.832
**Leptin/adiponectin ratio**	Birth weight z-score		0.32	0.82	0.695
**Leptin/ghrelin ratio**	Birth weight z-score		−0.60	1.87	0.750

Data are presented as Spearman’s correlation coefficients (ρ), regression coefficients (β), or number (percentage), as appropriate. Linear regression models were adjusted for age, sex, and study group. *p*-values for categorical comparisons were calculated using Fisher’s exact test where appropriate.

**Table 4:** Catch-up growth and cardiometabolic outcomes in the FGR group. (**A**) Categorical outcomes by catch-up growth status; (**B**) continuous outcomes by catch-up growth status; (**C**) adjusted association of ΔZ with selected outcomes (linear regression; adjusted for age and sex); (**D**) logistic regression (ΔZ → WC ≥ P90; adjusted for age and sex).

(**A**)					
**Outcome**	**Level**	**No Catch-Up (*n* = 6)**	**Catch-Up (*n* = 34)**	**Test**	***p*-Value**
Weight status	Normal	6 (100.0%)	20 (58.8%)	Fisher	0.074
	Overweight/Obesity	0 (0.0%)	14 (41.2%)		
WC P > 90th	No	6 (100.0%)	27 (79.4%)	Fisher	0.567
	Yes	0 (0.0%)	7 (20.6%)		
WHtR > 0.5	No	6 (100.0%)	31 (91.2%)	Fisher	1.000
	Yes	0 (0.0%)	3 (8.8%)		
High Glucose	No	6 (100.0%)	34 (100.0%)	Fisher	1.000
sBP ≥ P.90	No	6 (100.0%)	32 (94.1%)	Fisher	1.000
	Yes	0 (0.0%)	2 (5.9%)		
dBP ≥ P.90	No	5 (83.3%)	31 (91.2%)	Fisher	0.493
	Yes	1 (16.7%)	3 (8.8%)		
(**B**)					
**Outcome**		**No Catch-Up (*n* = 6)**	**Catch-Up (*n* = 34)**		***p*-Value**
BMI (kg/m^2^)		16.815 (2.720)	21.570 (5.525)		**0.020**
BMI *Z*-score		−1.015 (0.982)	0.530 (1.545)		**0.005**
(**C**)					
**Outcome**			**β Per 1-Unit ΔZ**	**SE**	***p*-Value**
BMI (kg/m^2^)			2.832	0.416	**<0.001**
BMI *Z*-score			0.713	0.107	**<0.001**
WC (cm)			5.334	0.771	**<0.001**
WHtR			0.022	0.007	**0.003**
sBP (mmHg)			4.113	1.148	**<0.001**
dBP (mmHg)			2.836	1.174	**0.021**
P.sBP			6.997	2.963	**0.024**
Leptin (ng/L)			0.22	0.092	**0.022**
Leptin/ghrelin ratio			2.33	1.059	**0.034**
(**D**)					
**Outcome**		**Events**	**OR Per 1-Unit ΔZ**	**95% CI**	***p*-Value**
WC ≥ P90		7	4.2	1.40–12.59	**0.010**

Data are presented as *n* (%) for categorical variables and median (IQR) for continuous variables. *p*-values were calculated using Fisher’s exact test (categorical comparisons) and the Wilcoxon rank-sum test (continuous comparisons). Bold *p*-value < 0.05 was considered statistically significant.

**Table 5:** Interaction between catch-up growth (ΔZ) and overweight/obesity on cardiometabolic outcomes in the FGR group. Adjusted linear regression models including an interaction term (ΔZ × OWOB).

Outcome	Effect	β	SE	*p*-Value
BMI (kg/m^2^)	Slope of ΔZ (Normal)	1.110	0.560	0.058
	Δ slope (OW/OB vs. Normal)	1.010	0.910	0.275
BMI *Z*-score	Slope of ΔZ (Normal)	0.430	0.140	**0.005**
	Δ slope (OW/OB vs. Normal)	−0.200	0.230	0.394
Waist circumference (cm)	Slope of ΔZ (Normal)	2.160	0.980	0.034
	Δ slope (OW/OB vs. Normal)	1.070	1.580	0.504
WHtR	Slope of ΔZ (Normal)	−0.002	0.008	0.844
	Δ slope (OW/OB vs. Normal)	−0.006	0.014	0.683
Systolic BP (mmHg)	Slope of ΔZ (Normal)	−0.660	1.720	0.704
	Δ slope (OW/OB vs. Normal)	8.130	2.780	**0.006**

Data are β coefficients from linear regression models adjusted for age and sex. The interaction term represents the difference in the ΔZ slope between overweight/obese and normal-weight children. Bold *p*-value < 0.05 was considered statistically significant.

**Table 6:** Independent predictors of cardiometabolic risk outcomes (multivariable logistic regression).

Outcome	*n*	Events	Predictor	OR	95% CI	*p*-Value
Dyslipidemia	80	35	Leptin/ghrelin ratio	0.80	0.36–1.02	0.383
			Age	0.86	0.62–1.18	0.348
			Leptin/adiponectin ratio	0.10	0.00–1.18	0.303
Elevated blood pressure	80	7	BMI (kg/m^2^)	1.28	1.09–1.60	0.007
			Age	0.72	0.40–1.29	0.253

Abbreviations: OR, odds ratio; CI, confidence interval; BMI, body mass index. Models were adjusted for age and sex. Predictor selection was performed using LASSO regularization. Bold *p*-value < 0.05 was considered statistically significant.

## Data Availability

The data presented in this study are available on request from the corresponding author due to privacy.

## Abbreviations

The following abbreviations are used in this manuscript:

FGR	Fetal growth restriction
WHO	World Health Organization
DOHaD	Developmental origins of health and disease
AGA	Appropriate for gestational age
SGA	Small for gestational age
SD	Standard deviation
BMI	Body mass index
WC	Waist circumference
WHtR	Waist-to-height ratio
BP	Blood pressure
HPA axis	Hypothalamic–pituitary–adrenal axis
MetS	Metabolic syndrome
ELISA	Enzyme-linked immunosorbent
